# The Multi-Attribute Group Decision-Making Method Based on Interval Grey Trapezoid Fuzzy Linguistic Variables

**DOI:** 10.3390/ijerph14121561

**Published:** 2017-12-13

**Authors:** Kedong Yin, Pengyu Wang, Xuemei Li

**Affiliations:** 1 School of Economics, Ocean University of China, Qingdao 266100, China; yinkedong@ouc.edu.cn (K.Y.); wangpengyu@stu.ouc.edu.cn (P.W.); 2 Ocean Development Research Institute, Major Research Base of Humanities and Social Sciences of Ministry of Education, Ocean University of China, Qingdao 266100, China

**Keywords:** interval grey trapezoid fuzzy linguistic variables, grey relation analysis, multi-attribute group decision making

## Abstract

With respect to multi-attribute group decision-making (MAGDM) problems, where attribute values take the form of interval grey trapezoid fuzzy linguistic variables (IGTFLVs) and the weights (including expert and attribute weight) are unknown, improved grey relational MAGDM methods are proposed. First, the concept of IGTFLV, the operational rules, the distance between IGTFLVs, and the projection formula between the two IGTFLV vectors are defined. Second, the expert weights are determined by using the maximum proximity method based on the projection values between the IGTFLV vectors. The attribute weights are determined by the maximum deviation method and the priorities of alternatives are determined by improved grey relational analysis. Finally, an example is given to prove the effectiveness of the proposed method and the flexibility of IGTFLV.

## 1. Introduction

Multi-attribute group decision-making (MAGDM) refers to the decision process of selecting optimal alternatives based on multiple attributes. In the field of modern decision science, MAGDM is a core focus of study. It is widely used in many fields such as engineering [1], technology [2], economy [3], management [4] and the military [5]. There are a number of uncertainties in these decision areas, and usually fuzziness and greyness are two important types of uncertainty. Fuzziness is the phenomenon whereby the decision maker cannot determine the category and state to which the decision object belongs. As with hot water and warm water, young people and middle-aged people, these concepts do not have clear boundaries. Greyness is the phenomenon whereby the decision-maker can only know the approximate range of information or part of the information, without knowing all of the information or the exact information. If MAGDM contains both fuzziness and greyness, it can be called grey fuzzy multi-attribute group decision-making (GFMAGDM).

After years of development, GFMAGDM has resulted in many significant achievements. Bu and Zhang [6] utilized the exact number to express the grey fuzzy number, and utilized different operators for the grey and fuzzy parts in order to reduce the loss of information. Often the decision-makers cannot express their fuzzy will using real numbers. Zhu et al. [7] utilized the interval number and real number to express fuzzy parts and grey parts, respectively. Compared to the real number, the interval number contains more information. So the grey part that is expressed by interval numbers reduces the loss of information. Wang and Wang [8] defined the grey and fuzzy parts as interval numbers, and the method of aggregating information utilized an ordered weighted averaging (OWA) operator. The triangular fuzzy number, the trapezoidal fuzzy number, and the three parameter interval grey numbers contain more information than the interval number. Hu et al. [9] brought triangular fuzzy numbers into fuzzy variables, and calculated the similarity between alternatives based on Euclidean distance. Cao and Shen [10] expanded the grey part into three parameter interval numbers, and proposed a method based on a projection model. Wang et al. [11] utilized trapezoidal fuzzy numbers to express the fuzzy parts, and sorted the alternatives by grey relational analysis. Linguistic terms as special fuzzy information have the advantages of easy availability, convenience, and so on. This can better express the wishes of the decision-makers [12]. Liu and Zhang [13] defined the concept of interval grey linguistic variables, the operational rules, the distance between interval grey linguistic variables, and proposed several weighted geometric aggregation operators. Liu [14] proposed several weighted aggregation operators based on interval grey linguistic variables. Jin et al. [15] proposed several weighted harmonic aggregation operators based on interval grey linguistic variables. Wei [16] utilized the 2-tuple linguistic terms to express the fuzzy part and proposed a multi-attribute decision-making (MADM) method based on grey relation analysis. Decision-makers sometimes cannot use exact linguistic terms to express their wishes. Ma et al. [17] proposed the concept of interval grey uncertain linguistic variables, and constructed several weighted arithmetic aggregation operators. Han et al. [18] utilized the concept of interval grey uncertain linguistic variables, and established three weighted geometric aggregation operators. In order to better express fuzzy information, Liu and Wang [19] defined the concept of generalized interval-valued trapezoidal fuzzy numbers, the operational rules, the distance between generalized interval-valued trapezoidal fuzzy numbers, and proposed several weighted geometric aggregation operators. The above research has improved a series of decision-making methods and decision models. Linguistic terms can be a good expression of the wishes of decision makers, and the interval grey number can be a good way to reduce the loss of information. However, sometimes the interval grey linguistic variables and interval grey uncertainty linguistic variables cannot meet the needs of decision-makers. In order to contain more information, this paper combines interval grey numbers with trapezoidal fuzzy linguistic variables.

In MAGDM theory, due to its characteristics of simplicity, practicability and operability, grey relational analysis is commonly used. Liu et al. [20] stated the basic principles and methods of grey relational decision making. Luo and Liu [21] explored the advantages and disadvantages of the classical grey relational decision method, extended grey relational model to interval grey number, and established the grey interval relation coefficient formula and the grey interval relative relation coefficient formula. Luo and Liu [22] utilized the maximum entropy criterion to study grey relational decision-making in incomplete information systems. Sun et al. [23] combined the Euclidean distance and the grey relational degree, and constructed a new degree of closeness to evaluate a program. Luo [24] defined the three-parameter interval grey number, and proposed a three-parameter grey interval relation method. Wang and Zhang [25] put forward a non-uniform grey relational decision method based on an analytic hierarchy process (AHP) and data envelopment analysis (DEA). According to the basic idea of the grey relational analysis method, Hu et al. [9] combined grey relation degree and Euclidean distance, and constructed an average similarity degree to evaluate the scheme. Jiang and Liu [26] proposed the formula of grey relational degree, which took the polygon area of the two adjacent points as the relation coefficient between the selected scheme and the ideal scheme. Yin et al. [27] proposed the concept of panel data dispersion and measurement methods, constructed the corresponding grey relational model based on dispersion of panel data (DPGRA) model. The grey relational decision method has also been improved according to a series of methods, such as Deng’s relation degree, comprehensive relation degree and area relation degree.

In the actual decision-making process, it is often impossible to obtain the exact weight of information, and the uncertainty of the weight will cause the uncertainty of the result. If there is almost no difference in the evaluation of alternatives under a certain attribute, this indicates that the attribute plays a minor role in the sorting process. Therefore, smaller weights should be given. On the contrary, if there are obvious differences in the evaluation of decision-makers, it indicates that this attribute plays an important role in the ordering process. Therefore, greater weight should be given [28,29]. In other words, from the perspective of sorting or selecting alternatives, the bigger deviation of attribute evaluation should be given the greater weight, and the smaller deviation of attribute evaluation should be given the smaller weight. In this paper, the attribute weights are also determined by the maximum deviation method. The method of determining the expert weights proposed by [30] is derived from the maximum deviation method. Unlike the maximum deviation method, the method of [30] is to minimize the deviation of group evaluation and individual evaluation of decision-makers. The deviation minimization of group evaluation and individual evaluation is equivalent to the similarity maximization. Therefore, this paper uses the projection value between IGTFLV (interval grey trapezoid fuzzy linguistic variables) vectors to express the similarity between group evaluation and individual evaluation. This paper also maximizes the total similarity to obtain expert weights.

The concept of IGTFLV is a generalization of the interval grey linguistic variables and interval grey uncertain linguistic variables. It not only contains more information, but also has a wider range of applications. Decision-makers can make more accurate evaluations. As expert evaluation is the form of IGTFLV, the attribute weights are determined by the maximum deviation method. However, it is difficult to use this method to determine expert weight when the expert evaluation is in the form of IGTFLV. Therefore, this paper adopts an equivalent method, that is, the method of maximum similarity. In Section 2, the necessary knowledge for this article is introduced. In Section 3, the concept of IGTFLV, the operational rules, the distance between IGTFLVs, and the projection formula between the two IGTFLV vectors, are defined. In Section 4, the expert weights are determined by using the maximum proximity method based on the projection values between the IGTFLV vectors. The attribute weights are determined by the maximum deviation method and the priorities of alternatives are determined by improved grey relational analysis. In Section 5, the group decision-making methods proposed in this paper are applied to evaluate the sewage-treatment capacity of enterprises, and the results of the two kinds of operators are discussed carefully.

## 2. Preliminaries

### 2.1. Grey Fuzzy Math

**Definition** **1.** *The grey fuzzy set in space* X *is defined as* $\underset{⊗}{\tilde{A}}=\left\{\left(x,\mu_{A}\left(x\right),\nu_{A}\left(x\right)\right)|x\in X\right\}$. *Using the set pair mode to express the grey fuzzy set the form is* $\underset{⊗}{\tilde{A}}=\left(\tilde{A},\underset{⊗}{A}\right)$. *The fuzzy part of* $\underset{⊗}{\tilde{A}}$ *is* $\tilde{A}=\left\{\left(x,\mu_{A}\left(x\right)\right)|x\in X\right\}$. *The grey part of* $\underset{⊗}{\tilde{A}}$ *is* $\underset{⊗}{A}=\left\{\left(x,\nu_{A}\left(x\right)\right)|x\in X\right\}$. *Therefore, the grey fuzzy set gathers the grey information and the fuzzy information, which can reflect the authenticity of the evaluation information*.

**Definition** **2.** *If two spaces* X={x} *and* Y={y} *are given, the grey fuzzy relationship in direct product space* X×Y *is* $\underset{⊗}{\tilde{R}}=\left\{\left(\left(x,y\right),\mu_{R}\left(x,y\right),\nu_{R}\left(x,y\right)\right)|x\in X,y\in Y\right\}$. *This grey fuzzy relationship can also be expressed in matrices:*

(1) $$\underset{⊗}{\tilde{R}}=\left[\begin{array}{cccc} \left(\mu_{11},\nu_{11}\right) & \left(\mu_{12},\nu_{12}\right) & \cdots & \left(\mu_{1n},\nu_{1n}\right)\\ \left(\mu_{21},\nu_{21}\right) & \left(\mu_{22},\nu_{22}\right) & \cdots & \left(\mu_{21},\nu_{21}\right)\\ \vdots & \vdots & \vdots & \vdots \\ \left(\mu_{m1},\nu_{m1}\right) & \left(\mu_{m2},\nu_{m2}\right) & \cdots & \left(\mu_{mn},\nu_{mn}\right) \end{array}\right]$$

*Similarly, this grey fuzzy relationship can also be expressed as* $\underset{⊗}{\tilde{R}}=\left(\tilde{R},\underset{⊗}{R}\right)$ *in the space* X×Y*, where the fuzzy relationship is* $\tilde{R}=\left\{\left(\left(x,y\right),\mu_{A}\left(x,y\right)\right)|x\in X,y\in Y\right\}$*, and the grey relationship is* $\underset{⊗}{R}=\left\{\left(\left(x,y\right),\nu_{A}\left(x,y\right)\right)|x\in X,y\in Y\right\}$ *[31,32,33,34]*.

### 2.2. Linguistic Terms Sets

Given that $S=\left(s_{0},s_{1},\cdot \cdot \cdot ,s_{l-1}\right)$ is a set of linguistic evaluations, the elements in the set are finite and orderly. The l in the set is an odd number. In practice, there are usually three, five, seven, and nine numbers to choose from. In this paper, l = 9. The meaning of each element in set S is as follows: $S=\left(s_{0},s_{1},s_{2},s_{3},s_{4},s_{5},s_{6},s_{7},s_{8}\right)$ = {extremely poor, very poor, poor, slightly worse, fair, slightly better, good, very good, extremely good} [35]. The linguistic evaluation set is ordered. The set also has the negation operator, maximum operator and minimum operator. This is no longer detailed here.

For any $S=\left(s_{0},s_{1},\cdot \cdot \cdot ,s_{l-1}\right)$, there is a strict, monotonically increasing relationship between element $s_{i}$ and its subscript i in the set [36,37]. Suppose the subscript function is $f:s_{i}=f\left(i\right)$. If i≺j then $s_{i}≺s_{j}$, and vice versa. The inverse function of the subscript function is $i=f^{-1}\left(s_{i}\right)$. If $s_{i}≺s_{j}$, then i≺j, and vice versa. The subscript function and its inverse functions are strictly monotonically functional. The discrete linguistic evaluation set $S=\left(s_{0},s_{1},\cdot \cdot \cdot ,s_{l-1}\right)$ will lose some evaluation information during the decision process. To minimize this loss, the set is extended into a continuous linguistic evaluation set $s=\left\{s_{\alpha }|\alpha \in R\right\}$. The extended linguistic scale still satisfies the above conditions and the operation rules remain the same [38].

(2) $$\beta s_{i}=s_{\beta \times i}$$

(3) $$s_{i}⊕s_{j}=s_{i+j}$$

(4) $$s_{i}⊕s_{j}=s_{j}⊕s_{i}$$

(5) $$\lambda \left(s_{i}⊕s_{j}\right)=\lambda s_{i}⊕\lambda s_{j}$$

(6) $$(\lambda_{1}+\lambda_{2})s_{i}=\lambda_{1}s_{i}⊕\lambda_{2}s_{j}$$

**Definition** **3.** *Suppose there are two linguistic variables* $s_{\alpha }$ *and* $s_{\beta }$*, then the distance between them is [39]:*

(7) $$d\left(s_{\alpha },s_{\beta }\right)=\left|\alpha -\beta \right|/\left(l-1\right)$$

## 3. Interval Grey Trapezoid Fuzzy Linguistic Variables

**Definition** **4.** *If the trapezoid fuzzy linguistic variable* $\left[s_{\alpha },s_{\beta },s_{\delta },s_{\gamma }\right]$ *is the fuzzy part and closed interval* $\left[g_{A}^{L},g_{A}^{U}\right]$ *is the grey part of the grey fuzzy number, then the grey fuzzy number* $\underset{⊗}{\tilde{A}}=\left(\tilde{A},\underset{⊗}{A}\right)$ *is called the interval grey trapezoid fuzzy linguistic variables, which is an improved form of linguistic variables and uncertain linguistic variables. When* $s_{\alpha }=s_{\beta }=s_{\delta }=s_{\gamma }$*, the trapezoid fuzzy linguistic variable becomes a linguistic variable. When* $s_{\alpha }=s_{\beta }<s_{\delta }=s_{\gamma }$*, the trapezoid fuzzy linguistic variable becomes an uncertain linguistic variable. Therefore, trapezoid fuzzy linguistic variables can express fuzzy information more efficiently. The grey part describes the uncertainty of the information. When the uncertainty of the information becomes larger, the grey degree becomes larger, since only greater greyscale can contain greater uncertainty. As a result, the information becomes more difficult to believe when the grey level becomes larger. On the contrary, when the credibility of the information becomes larger, the grey degree becomes smaller, and the uncertainty can be described using a smaller grey degree. Thus, when the grey scale becomes smaller, the information becomes easier to believe.*

### 3.1. Operational Rules of IGTFLVs

It is supposed that $\underset{⊗}{\tilde{A}}=\left(\left[s_{\alpha_{1}},s_{\beta_{1}},s_{\delta_{1}},s_{\gamma_{1}}\right],\left[g_{A}^{L},g_{A}^{U}\right]\right)$, $\underset{⊗}{\tilde{B}}=\left(\left[s_{\alpha_{2}},s_{\beta_{2}},s_{\delta_{2}},s_{\gamma_{2}}\right],\left[g_{B}^{L},g_{B}^{U}\right]\right)$ and $\underset{⊗}{\tilde{C}}=\left(\left[s_{\alpha_{3}},s_{\beta_{3}},s_{\delta_{3}},s_{\gamma_{3}}\right],\left[g_{C}^{L},g_{C}^{U}\right]\right)$. According to the above definitions, the operating rules and the extension principle of linguistic variables, the algorithm of IGTFLVs is defined as follows:

(8) $$\underset{⊗}{\tilde{A}}+\underset{⊗}{\tilde{B}}=\left(\left[s_{\alpha_{1}+\alpha_{2}},s_{\beta_{1}+\beta_{2}},s_{\delta_{1}+\delta_{2}},s_{\gamma_{1}+\gamma_{2}}\right],\left[\left(1-\left(1-g_{A}^{L}\right)\times \left(1-g_{B}^{L}\right)\right),\left(1-\left(1-g_{A}^{U}\right)\times \left(1-g_{B}^{U}\right)\right)\right]\right)$$

(9) $$\underset{⊗}{\tilde{A}}-\underset{⊗}{\tilde{B}}=\left(\left[s_{\alpha_{1}-\gamma_{2}},s_{\beta_{1}-\delta_{2}},s_{\delta_{1}-\beta_{2}},s_{\gamma_{1}-\alpha_{2}}\right],\left[\left(1-\left(1-g_{A}^{L}\right)\times \left(1-g_{B}^{L}\right)\right),\left(1-\left(1-g_{A}^{U}\right)\times \left(1-g_{B}^{U}\right)\right)\right]\right)$$

(10) $$\underset{⊗}{\tilde{A}}\times \underset{⊗}{\tilde{B}}=\left(\left[s_{\alpha_{1}\times \alpha_{2}},s_{\beta_{1}\times \beta_{2}},s_{\delta_{1}\times \delta_{2}},s_{\gamma_{1}\times \gamma_{2}}\right],\left[\left(1-\left(1-g_{A}^{L}\right)\times \left(1-g_{B}^{L}\right)\right),\left(1-\left(1-g_{A}^{U}\right)\times \left(1-g_{B}^{U}\right)\right)\right]\right)$$

(11) $$\underset{⊗}{\tilde{A}}÷\underset{⊗}{\tilde{B}}=\left(\left[s_{\alpha_{1}/\gamma_{2}},s_{\beta_{1}/\delta_{2}},s_{\delta_{1}/\beta_{2}},s_{\gamma_{1}/\alpha_{2}}\right],\left[\left(1-\left(1-g_{A}^{L}\right)\times \left(1-g_{B}^{L}\right)\right),\left(1-\left(1-g_{A}^{U}\right)\times \left(1-g_{B}^{U}\right)\right)\right]\right)$$

(12) $$k\underset{⊗}{\tilde{A}}=\left(\left[s_{k\alpha_{1}},s_{k\beta_{1}},s_{k\delta_{1}},s_{k\gamma_{1}}\right],\left[g_{A}^{L},g_{A}^{U}\right]\right)$$

(13) $${\left(\underset{⊗}{\tilde{A}}\right)}^{k}=\left(\left[s_{\alpha_{1}^{k}},s_{\beta_{1}^{k}},s_{\delta_{1}^{k}},s_{\gamma_{1}^{k}}\right],\left[\left(1-{\left(1-g_{A}^{L}\right)}^{k}\right),\left(1-{\left(1-g_{A}^{U}\right)}^{k}\right)\right]\right)$$

It is easy to prove that the IGTFLVs satisfy the computational properties such as the distribution law, union law and exchange law.

(14) $$\underset{⊗}{\tilde{A}}+\underset{⊗}{\tilde{B}}=\underset{⊗}{\tilde{B}}+\underset{⊗}{\tilde{A}}$$

(15) $$\underset{⊗}{\tilde{A}}\times \underset{⊗}{\tilde{B}}=\underset{⊗}{\tilde{B}}\times \underset{⊗}{\tilde{A}}$$

(16) $$\underset{⊗}{\tilde{A}}+\underset{⊗}{\tilde{B}}+\underset{⊗}{\tilde{C}}=\underset{⊗}{\tilde{A}}+\left(\underset{⊗}{\tilde{B}}+\underset{⊗}{\tilde{C}}\right)$$

(17) $$\underset{⊗}{\tilde{A}}\times \underset{⊗}{\tilde{B}}\times \underset{⊗}{\tilde{C}}=\underset{⊗}{\tilde{A}}\times \left(\underset{⊗}{\tilde{B}}\times \underset{⊗}{\tilde{C}}\right)$$

(18) $$\underset{⊗}{\tilde{A}}\times \left(\underset{⊗}{\tilde{B}}+\underset{⊗}{\tilde{C}}\right)=\underset{⊗}{\tilde{A}}\times \underset{⊗}{\tilde{B}}+\underset{⊗}{\tilde{A}}\times \underset{⊗}{\tilde{C}}$$

(19) $$\left(\lambda_{1}+\lambda_{2}\right)\underset{⊗}{\tilde{A}}=\lambda_{1}\underset{⊗}{\tilde{A}}+\lambda_{2}\underset{⊗}{\tilde{A}}$$

### 3.2. Hamming Distance of IGTFLVs

**Definition** **5.** *According to the definition of the distance of the grey fuzzy number, the grey fuzzy number needs to satisfy three properties. Therefore, we define the IGTFLV as also needing to meet these three properties. Let $\underset{⊗}{\tilde{A}}$, $\underset{⊗}{\tilde{B}}$ and $\underset{⊗}{\tilde{C}}$*be IGTFLVs.*$\underset{⊗}{\tilde{Z}}$ be the set of IGTFLVs, f be the mapping, $f:\underset{⊗}{\tilde{Z}}\times \underset{⊗}{\tilde{Z}}\to \underset{⊗}{\tilde{R}}$. If $d\left(\underset{⊗}{\tilde{A}},\underset{⊗}{\tilde{B}}\right)$, and satisfied the following formula:*

(20) $$0\leq d\left(\underset{⊗}{\tilde{A}},\underset{⊗}{\tilde{B}}\right)\leq 1,\;d\left(\underset{⊗}{\tilde{A}},\underset{⊗}{\tilde{A}}\right)=0$$

(21) $$d\left(\underset{⊗}{\tilde{A}},\underset{⊗}{\tilde{B}}\right)=d\left(\underset{⊗}{\tilde{B}},\underset{⊗}{\tilde{A}}\right)$$

(22) $$d\left(\underset{⊗}{\tilde{A}},\underset{⊗}{\tilde{B}}\right)+d\left(\underset{⊗}{\tilde{B}},\underset{⊗}{\tilde{C}}\right)\geq d\left(\underset{⊗}{\tilde{A}},\underset{⊗}{\tilde{C}}\right)$$

*then the Hamming distance* $d\left(\underset{⊗}{\tilde{A}},\underset{⊗}{\tilde{B}}\right)$ *is called the distance between IGTFLV* $\underset{⊗}{\tilde{A}}$ *and* $\underset{⊗}{\tilde{B}}$.

**Definition** **6.** *Let* $\underset{⊗}{\tilde{A}}=\left(\left[s_{\alpha_{1}},s_{\beta_{1}},s_{\delta_{1}},s_{\gamma_{1}}\right],\left[g_{A}^{L},g_{A}^{U}\right]\right)$ *and* $\underset{⊗}{\tilde{B}}=\left(\left[s_{\alpha_{2}},s_{\beta_{2}},s_{\delta_{2}},s_{\gamma_{2}}\right],\left[g_{B}^{L},g_{B}^{U}\right]\right)$ *be the IGTFLVs, then the Hamming distance* $d\left(\underset{⊗}{\tilde{A}},\underset{⊗}{\tilde{B}}\right)$ *is defined as follows:*

(23) $$\begin{array}{c} d\left(\underset{⊗}{\tilde{A}},\underset{⊗}{\tilde{B}}\right)=\frac{1}{8\left(l-1\right)}(\left|\alpha_{1}\left(1-g_{A}^{L}\right)-\alpha_{2}\left(1-g_{B}^{L}\right)\right|+\left|\alpha_{1}\left(1-g_{A}^{U}\right)-\alpha_{2}\left(1-g_{B}^{U}\right)\right|+\left|\beta_{1}\left(1-g_{A}^{L}\right)-\beta_{2}\left(1-g_{B}^{L}\right)\right|\\ +\left|\beta_{1}\left(1-g_{A}^{U}\right)-\beta_{2}\left(1-g_{B}^{U}\right)\right|+\left|\delta_{1}\left(1-g_{A}^{L}\right)-\delta_{2}\left(1-g_{B}^{L}\right)\right|+\left|\delta_{1}\left(1-g_{A}^{U}\right)-\delta_{2}\left(1-g_{B}^{U}\right)\right|\\ +\left|\gamma_{1}\left(1-g_{A}^{L}\right)-\gamma_{2}\left(1-g_{B}^{L}\right)\right|+\left|\gamma_{1}\left(1-g_{A}^{U}\right)-\gamma_{2}\left(1-g_{B}^{U}\right)\right|) \end{array}$$

**Proof.**  The properties $0\leq d\left(\underset{⊗}{\tilde{A}},\underset{⊗}{\tilde{B}}\right)\leq 1,d\left(\underset{⊗}{\tilde{A}},\underset{⊗}{\tilde{A}}\right)=0$ and $d\left(\underset{⊗}{\tilde{A}},\underset{⊗}{\tilde{B}}\right)=d\left(\underset{⊗}{\tilde{B}},\underset{⊗}{\tilde{A}}\right)$ are easy to prove, which is no longer extended here. For any IGTFLVs $\underset{⊗}{\tilde{C}}=\left(\left[s_{\alpha_{3}},s_{\beta_{3}},s_{\delta_{3}},s_{\gamma_{3}}\right],\left[g_{C}^{L},g_{C}^{U}\right]\right)$, we can get:

(24) $$\begin{array}{l} d\left(\underset{⊗}{\tilde{A}},\underset{⊗}{\tilde{C}}\right)=\frac{1}{8\left(l-1\right)}(\left|\alpha_{1}\left(1-g_{A}^{L}\right)-\alpha_{3}\left(1-g_{C}^{L}\right)\right|+\left|\alpha_{1}\left(1-g_{A}^{U}\right)-\alpha_{3}\left(1-g_{C}^{U}\right)\right|+\left|\beta_{1}\left(1-g_{A}^{L}\right)-\beta_{3}\left(1-g_{C}^{L}\right)\right|\\ +\left|\beta_{1}\left(1-g_{A}^{U}\right)-\beta_{3}\left(1-g_{C}^{U}\right)\right|+\left|\delta_{1}\left(1-g_{A}^{L}\right)-\delta_{3}\left(1-g_{C}^{L}\right)\right|+\left|\delta_{1}\left(1-g_{A}^{U}\right)-\delta_{3}\left(1-g_{C}^{U}\right)\right|\\ +\left|\gamma_{1}\left(1-g_{A}^{L}\right)-\gamma_{3}\left(1-g_{C}^{L}\right)\right|+\left|\gamma_{1}\left(1-g_{A}^{U}\right)-\gamma_{3}\left(1-g_{C}^{U}\right)\right|)\\ \leq \frac{1}{8\left(l-1\right)}(\left|\alpha_{1}\left(1-g_{A}^{L}\right)-\alpha_{2}\left(1-g_{B}^{L}\right)\right|+\left|\alpha_{2}\left(1-g_{B}^{L}\right)-\alpha_{3}\left(1-g_{C}^{L}\right)\right|+\left|\alpha_{1}\left(1-g_{A}^{U}\right)-\alpha_{2}\left(1-g_{B}^{U}\right)\right|\\ +\left|\alpha_{2}\left(1-g_{B}^{U}\right)-\alpha_{3}\left(1-g_{C}^{U}\right)\right|+\left|\beta_{1}\left(1-g_{A}^{L}\right)-\beta_{2}\left(1-g_{B}^{L}\right)\right|+\left|\beta_{2}\left(1-g_{B}^{L}\right)-\beta_{3}\left(1-g_{C}^{L}\right)\right|+\\ \left|\beta_{1}\left(1-g_{A}^{U}\right)-\beta_{2}\left(1-g_{B}^{U}\right)\right|+\left|\beta_{2}\left(1-g_{B}^{U}\right)-\beta_{3}\left(1-g_{C}^{U}\right)\right|+\left|\delta_{1}\left(1-g_{A}^{L}\right)-\delta_{2}\left(1-g_{B}^{L}\right)\right|+\\ \left|\delta_{2}\left(1-g_{B}^{L}\right)-\delta_{3}\left(1-g_{C}^{L}\right)\right|+\left|\delta_{1}\left(1-g_{A}^{U}\right)-\delta_{2}\left(1-g_{B}^{U}\right)\right|+\left|\delta_{2}\left(1-g_{B}^{U}\right)-\delta_{3}\left(1-g_{C}^{U}\right)\right|+\\ \left|\gamma_{1}\left(1-g_{A}^{L}\right)-\gamma_{2}\left(1-g_{B}^{L}\right)\right|+\left|\gamma_{2}\left(1-g_{B}^{L}\right)-\gamma_{3}\left(1-g_{C}^{L}\right)\right|+\left|\gamma_{1}\left(1-g_{A}^{U}\right)-\gamma_{2}\left(1-g_{B}^{U}\right)\right|+\\ \left|\gamma_{2}\left(1-g_{B}^{U}\right)-\gamma_{3}\left(1-g_{C}^{U}\right)\right|)=d\left(\underset{⊗}{\tilde{A}},\underset{⊗}{\tilde{B}}\right)+d\left(\underset{⊗}{\tilde{B}},\underset{⊗}{\tilde{C}}\right) \end{array}$$

If $g_{A}^{L}=g_{A}^{U}=g_{B}^{L}=g_{B}^{U}=0$, the evaluation information is completely believable and absolutely reliable. Then Formula (23) will degenerate into the form of Formula (25):

(25) $$d\left(\underset{⊗}{\tilde{A}},\underset{⊗}{\tilde{B}}\right)=\frac{1}{4\left(l-1\right)}\left(\left|\alpha_{1}-\alpha_{2}\right|+\left|\beta_{1}-\beta_{2}\right|+\left|\delta_{1}-\delta_{2}\right|+\left|\gamma_{1}-\gamma_{2}\right|\right)$$

**Example.**  *Let* $\underset{⊗}{\tilde{A}}=\left(\left[s_{2},s_{4},s_{6},s_{8}\right],\left[0.3,0.5\right]\right)$ *and* $\underset{⊗}{\tilde{B}}=\left(\left[s_{1},s_{3},s_{5},s_{7}\right],\left[0.4,0.6\right]\right)$ *be the IGTFLVs*.

(26) $$\begin{array}{c} d\left(\underset{⊗}{\tilde{A}},\underset{⊗}{\tilde{B}}\right)=\frac{1}{8\times 8}(\left|2\left(1-0.3\right)-1\left(1-0.4\right)\right|+\left|2\left(1-0.5\right)-1\left(1-0.6\right)\right|+\left|4\left(1-0.3\right)-3\left(1-0.4\right)\right|\\ +\left|4\left(1-0.5\right)-3\left(1-0.6\right)\right|+\left|6\left(1-0.3\right)-5\left(1-0.4\right)\right|+\left|6\left(1-0.5\right)-5\left(1-0.6\right)\right|\\ +\left|8\left(1-0.3\right)-7\left(1-0.4\right)\right|+\left|8\left(1-0.5\right)-7\left(1-0.6\right)\right|)=0.125 \end{array}$$

### 3.3. IGTFLV Vector

**Definition** **7.** *The expression of the IGTFLV vector is:*

(27) $$A=\left(\underset{⊗j}{\tilde{A}}\right)=\left(\left(\left[s_{\alpha_{1}},s_{\beta_{1}},s_{\delta_{1}},s_{\gamma_{1}}\right],\left[g_{1}^{L},g_{1}^{U}\right]\right),\left(\left[s_{\alpha_{2}},s_{\beta_{2}},s_{\delta_{2}},s_{\gamma_{2}}\right],\left[g_{2}^{L},g_{2}^{U}\right]\right),\cdots ,\left(\left[s_{\alpha_{n}},s_{\beta_{n}},s_{\delta_{n}},s_{\gamma_{n}}\right],\left[g_{n}^{L},g_{n}^{U}\right]\right)\right)$$

*The magnitude of the vector* A *is*

(28) $$\left|A\right|=\sqrt{\sum_{j=1}^{n}\left(\left({\left(1-g_{j}^{L}\right)}^{2}+{\left(1-g_{j}^{U}\right)}^{2}\right)\times \left(\alpha_{j}^{2}+\beta_{j}^{2}+\delta_{j}^{2}+\gamma_{j}^{2}\right)\right)}$$

**Definition** **8.** *Let* A *and* $A^{\prime }$ *be IGTFLV vectors. Then the cosine of the angle between the two vectors is:*

(29) $$\cos\left(A,A^{\prime }\right)=\frac{\sum_{j=1}^{n}\left(\left(\left(1-g_{j}^{L}\right)\left(1-g_{j}^{L^{\prime }}\right)+\left(1-g_{j}^{U}\right)\left(1-g_{j}^{U^{\prime }}\right)\right)\left(\alpha_{j}{\alpha^{\prime }}_{j}+\beta_{j}{\beta^{\prime }}_{j}+\delta_{j}{\delta^{\prime }}_{j}+\gamma_{j}{\gamma^{\prime }}_{j}\right)\right)}{\sqrt{\sum_{j=1}^{n}\left(\left({\left(1-g_{j}^{L}\right)}^{2}+{\left(1-g_{j}^{U}\right)}^{2}\right)\times \left(\alpha_{j}^{2}+\beta_{j}^{2}+\delta_{j}^{2}+\gamma_{j}^{2}\right)\right)}\sqrt{\sum_{j=1}^{n}\left(\left({\left(1-g_{j}^{L^{\prime }}\right)}^{2}+{\left(1-g_{j}^{U^{\prime }}\right)}^{2}\right)\times \left({\alpha^{\prime }}_{j}^{2}+{\beta^{\prime }}_{j}^{2}+{\delta^{\prime }}_{j}^{2}+{\gamma^{\prime }}_{j}^{2}\right)\right)}}$$

*The larger the Angle cosine, the closer the* A *and* $A^{\prime }$*, the higher the degree of closeness*.

**Definition** **9.** *Let* A *and* $A^{\prime }$ *be IGTFLV vectors. Then the projection of* A *on to* $A^{\prime }$ *is*

(30) $${\mathrm{Prj}}_{A}\;A^{\prime }=\frac{\sum_{j=1}^{n}\left(\left(\left(1-g_{j}^{L}\right)\left(1-g_{j}^{L^{\prime }}\right)+\left(1-g_{j}^{U}\right)\left(1-g_{j}^{U^{\prime }}\right)\right)\left(\alpha_{j}{\alpha^{\prime }}_{j}+\beta_{j}{\beta^{\prime }}_{j}+\delta_{j}{\delta^{\prime }}_{j}+\gamma_{j}{\gamma^{\prime }}_{j}\right)\right)}{\sqrt{\sum_{j=1}^{n}\left(\left({\left(1-g_{j}^{L}\right)}^{2}+{\left(1-g_{j}^{U}\right)}^{2}\right)\times \left(\alpha_{j}^{2}+\beta_{j}^{2}+\delta_{j}^{2}+\gamma_{j}^{2}\right)\right)}}$$

*Obviously, the larger the* ${\mathrm{Prj}}_{A}\;A^{\prime }$ *, the closer the* A *and* $A^{\prime }$*, and the higher the degree of closeness*.

## 4. MAGDM Method Based on Grey Relational Analysis

### 4.1. Problem Description

**Definition** **10.** *In a MAGDM process,* $E=\left\{e_{k}|k=1,2,\cdots ,p\right\}$ *is a set of experts,* $A=\left\{A_{i}|i=1,2,\cdots ,m\right\}$ *is an alternative set, and* $C=\left\{C_{j}|j=1,2,\cdots ,n\right\}$ *is a set of attributes. The attribute set of alternative* $A_{i}$ *is* $C_{j}$*, and the attribute value* $\underset{⊗ijk}{\tilde{A}}$ *is expressed as* $\underset{⊗ijk}{\tilde{A}}=\left(\left[s_{\alpha_{ijk}},s_{\beta_{ijk}},s_{\delta_{ijk}},s_{\gamma_{ijk}}\right],\left[g_{ijk}^{L},g_{ijk}^{U}\right]\right)$. *According to the actual situation and through scientific conclusions, expert* $e_{k}$ *gives the decision matrix* $\underset{⊗k}{\tilde{A}}={\left[\underset{⊗ijk}{\tilde{A}}\right]}_{m\times n}$ *, where* $\underset{⊗ijk}{\tilde{A}}=\left(\left[s_{\alpha_{ijk}},s_{\beta_{ijk}},s_{\delta_{ijk}},s_{\gamma_{ijk}}\right],\left[g_{ijk}^{L},g_{ijk}^{U}\right]\right)$. *The attribute value given by expert* $e_{k}$ *contains the fuzzy part and the grey part, which are represented by* $\left[s_{\alpha_{ijk}},s_{\beta_{ijk}},s_{\delta_{ijk}},s_{\gamma_{ijk}}\right]$ *and* $\left[g_{ijk}^{L},g_{ijk}^{U}\right]$*, respectively*.

$w=\left(w_{1},w_{2},\cdots ,w_{n}\right)$ *is the attributes weight, where* $\sum_{j=1}^{n}w_{k}=1$. $\lambda =\left(\lambda_{1},\lambda_{2},\cdot \cdot \cdot ,\lambda_{p}\right)$ *is the experts’ weight, where* $\sum_{k=1}^{p}\lambda_{k}=1$. *These two kinds of weights are unknown in this article. According to the conditions we have obtained, we can sort out the advantages and disadvantages of alternatives*.

### 4.2. The Decision-Making Steps

#### 4.2.1. Determine the Expert Weight

The evaluation of the attribute $C_{j}$ of the expert $e_{k}$ for the alternative $A_{i}$ is $\underset{⊗ijk}{\tilde{A}}$ and the decision matrix given by expert $e_{k}$ is $\underset{⊗k}{\tilde{A}}$. Assume that the expert weight is $\lambda =\left(\lambda_{1},\lambda_{2},\cdot \cdot \cdot ,\lambda_{p}\right)$. Then, the group decision matrix $\underset{⊗}{\tilde{X}}$ is obtained by using the following formula:

Let $\underset{⊗}{\tilde{X}}={\left[\underset{⊗ij}{\tilde{X}}\right]}_{m\times n}$, and $\underset{⊗ij}{\tilde{X}}=\left(\left(s_{\alpha_{ij}^{X}},s_{\beta_{ij}^{X}},s_{\delta_{ij}^{X}},s_{\gamma_{ij}^{X}}\right),\left[g_{ij}^{L},g_{ij}^{U}\right]\right)$. Using the weighted arithmetic average algorithm, we can get $\underset{⊗ij}{\tilde{X}}=\sum_{k=1}^{p}\left(\lambda_{k}\underset{⊗ijk}{\tilde{A}}\right)$, where:

(31) $$s_{\alpha_{ij}^{X}}=\sum_{k=1}^{p}\left(\lambda_{k}s_{\alpha_{ijk}}\right),\;s_{\beta_{ij}^{X}}=\sum_{k=1}^{p}\left(\lambda_{k}s_{\beta_{ijk}}\right),\;s_{\delta_{ij}^{X}}=\sum_{k=1}^{p}\left(\lambda_{k}s_{\delta_{ijk}}\right),\;s_{\gamma_{ij}^{X}}=\sum_{k=1}^{p}\left(\lambda_{k}s_{\gamma_{ijk}}\right)$$

(32) $$\left[g_{ij}^{L},g_{ij}^{U}\right]=\left[\left(1-\prod_{k=1}^{p}\left(1-g_{ijk}^{L}\right)\right),\left(1-\prod_{k=1}^{p}\left(1-g_{ijk}^{U}\right)\right)\right]$$

In order to simplify the expression, let $g_{ijk}=\left(\left(1-g_{ijk}^{L}\right)\left(\prod_{k=1}^{p}\left(1-g_{ijk}^{L}\right)\right)+\left(1-g_{ijk}^{U}\right)\left(\prod_{k=1}^{p}\left(1-g_{ijk}^{U}\right)\right)\right)$.

Therefore, the evaluation vector of expert $e_{k}$ for the alternative $A_{i}$ is $A_{ik}$. The magnitude of $A_{ik}$ is $\left|A_{ik}\right|=\sqrt{\sum_{j=1}^{n}\left(\left({\left(1-g_{ijk}^{L}\right)}^{2}+{\left(1-g_{ijk}^{U}\right)}^{2}\right)\times \left(\alpha_{ijk}^{2}+\beta_{ijk}^{2}+\delta_{ijk}^{2}+\gamma_{ijk}^{2}\right)\right)}$.

Then the projection of $A_{ik}$ on to $X_{i}$ is

(33) $${\mathrm{Prj}}_{A_{ik}}\;X_{i}=\frac{\sum_{j=1}^{n}\left(g_{ijk}\left(\alpha_{ijk}\left(\sum_{k=1}^{p}\lambda_{k}\alpha_{ijk}\right)+\beta_{ijk}\left(\sum_{k=1}^{p}\lambda_{k}\beta_{ijk}\right)+\delta_{ijk}\left(\sum_{k=1}^{p}\lambda_{k}\delta_{ijk}\right)+\gamma_{ijk}\left(\sum_{k=1}^{p}\lambda_{k}\gamma_{ijk}\right)\right)\right)}{\left|A_{ik}\right|}$$

Now, the maximization of total similarity leads to the following optimization problem:

(34) $$\begin{array}{l} \max\;\sum_{i=1}^{m}\sum_{k=1}^{p}{\mathrm{Prj}}_{A_{ik}}X_{i}\\ s.t.\;\left\{ \begin{array}{c} \sum_{k=1}^{p}\lambda_{k}=1\\ \lambda_{k}\geq 0,k=1,2,\cdots ,p \end{array}\right. \end{array}$$

This is a convex function optimization problem with equality and inequality constraints, so it can be solved by the Lagrange multiplier method with the Karush-Kuhn-Tucker (KKT) conditions. Lagrange’s function can be constructed as follows:

(35) $$\begin{array}{cc} L\left(\lambda_{1},\lambda_{2},\cdots ,\lambda_{p},\eta ,\xi_{1},\xi_{2},\cdots ,\xi_{p}\right)= & \sum_{i=1}^{m}\sum_{k=1}^{p}\frac{\sum_{j=1}^{n}\left(g_{ijk}\left(\alpha_{ijk}\left(\sum_{k=1}^{p}\lambda_{k}\alpha_{ijk}\right)+\beta_{ijk}\left(\sum_{k=1}^{p}\lambda_{k}\beta_{ijk}\right)+\delta_{ijk}\left(\sum_{k=1}^{p}\lambda_{k}\delta_{ijk}\right)+\gamma_{ijk}\left(\sum_{k=1}^{p}\lambda_{k}\gamma_{ijk}\right)\right)\right)}{\left|A_{ik}\right|}\\ & +\eta \left(\sum_{k=1}^{p}\lambda_{k}-1\right)+\sum_{k=1}^{p}\xi_{k}\lambda_{k},\forall \xi_{k}\geq 0 \end{array}$$

Solution of equations:

(36) $$\left\{ \begin{array}{c} \frac{\partial L\left(\lambda_{1},\lambda_{2},\cdots ,\lambda_{p},\eta ,\xi_{1},\xi_{2},\cdots ,\xi_{p}\right)}{\partial \lambda_{1}}=\sum_{i=1}^{m}\sum_{k=1}^{p}\frac{\sum_{j=1}^{n}\left(g_{ijk}\left(\alpha_{ijk}\alpha_{ij1}+\beta_{ijk}\beta_{ij1}+\delta_{ijk}\delta_{ij1}+\gamma_{ijk}\gamma_{ij1}\right)\right)}{\left|A_{ik}\right|}\lambda_{1}+\eta +\xi_{1}=0\\ \frac{\partial L\left(\lambda_{1},\lambda_{2},\cdots ,\lambda_{p},\eta ,\xi_{1},\xi_{2},\cdots ,\xi_{p}\right)}{\partial \lambda_{2}}=\sum_{i=1}^{m}\sum_{k=1}^{p}\frac{\sum_{j=1}^{n}\left(g_{ijk}\left(\alpha_{ijk}\alpha_{ij2}+\beta_{ijk}\beta_{ij2}+\delta_{ijk}\delta_{ij2}+\gamma_{ijk}\gamma_{ij2}\right)\right)}{\left|A_{ik}\right|}\lambda_{2}+\eta +\xi_{2}=0\\ \vdots \;\\ \frac{\partial L\left(\lambda_{1},\lambda_{2},\cdots ,\lambda_{p},\eta ,\xi_{1},\xi_{2},\cdots ,\xi_{p}\right)}{\partial \lambda_{p}}=\sum_{i=1}^{m}\sum_{k=1}^{p}\frac{\sum_{j=1}^{n}\left(g_{ijk}\left(\alpha_{ijk}\alpha_{ijp}+\beta_{ijk}\beta_{ijp}+\delta_{ijk}\delta_{ijp}+\gamma_{ijk}\gamma_{ijp}\right)\right)}{\left|A_{ik}\right|}\lambda_{p}+\eta +\xi_{p}=0\\ \sum_{k=1}^{p}\lambda_{k}-1=0,\xi_{1}\lambda_{1}=0,\xi_{2}\lambda_{2}=0,\cdots ,\xi_{p}\lambda_{p}=0 \end{array}\right.$$

The results are the expert weights $\lambda =\left(\lambda_{1},\lambda_{2},\cdot \cdot \cdot ,\lambda_{p}\right)$. Therefore, the group decision matrix $\underset{⊗}{\tilde{X}}={\left[\underset{⊗ij}{\tilde{X}}\right]}_{m\times n}$ can be obtained by substituting the expert weights into Equations (24) and (25).

#### 4.2.2. Determine the Attribute Weights

For the attribute $C_{j}$, specifies that $D_{ij}\left(w_{j}\right)=\sum_{l=1}^{m}d\left(\underset{⊗ij}{\tilde{X}},\underset{⊗lj}{\tilde{X}}\right)w_{j}$ represents the total deviation of alternative $A_{i}$ from all other alternatives.

Then, for all attributes $D\left(w_{j}\right)=\sum_{j=1}^{n}\sum_{i=1}^{m}D_{ij}\left(w_{j}\right)=\sum_{j=1}^{n}\sum_{i=1}^{m}\sum_{l=1}^{m}d\left(\underset{⊗ij}{\tilde{X}},\underset{⊗lj}{\tilde{X}}\right)w_{j}$ represents the total deviation.

Now, the maximization of total deviation leads to the following optimization problem:

(37) $$\begin{array}{l} \max\;D\left(w_{j}\right)=\sum_{j=1}^{n}\sum_{i=1}^{m}D_{ij}\left(w_{j}\right)=\sum_{j=1}^{n}\sum_{i=1}^{m}\sum_{l=1}^{m}d\left(\underset{⊗ij}{\tilde{X}},\underset{⊗lj}{\tilde{X}}\right)w_{j}\\ s.t.\;\left\{ \begin{array}{c} \sum_{j=1}^{n}w_{j}^{2}=1\\ w_{j}\geq 0,j=1,2,\cdots ,n \end{array}\right. \end{array}$$

This is a convex function optimization problem with equality and inequality constraints, so it can be solved by the Lagrange multiplier method with KKT conditions. Lagrange’s function can be constructed as follows:

(38) $$L\left(w_{1},w_{2},\cdots ,w_{n},\eta ,\xi_{1},\xi_{2},\cdots ,\xi_{n}\right)=\sum_{j=1}^{n}\sum_{i=1}^{m}\sum_{l=1}^{m}d\left(\underset{⊗ij}{\tilde{X}},\underset{⊗lj}{\tilde{X}}\right)w_{j}+\eta \left(\sum_{j=1}^{n}w_{j}^{2}-1\right)+\sum_{j=1}^{n}\xi_{j}w_{j},\;\forall \xi_{j}\geq 0$$

The solution of this Lagrange’s function is consistent with the above method.

(39) $$\left\{ \begin{array}{c} \frac{\partial L\left(w_{1},w_{2},\cdots ,w_{n},\eta ,\xi_{1},\xi_{2},\cdots ,\xi_{n}\right)}{\partial w_{1}}=\sum_{i=1}^{m}\sum_{l=1}^{m}d\left(\underset{⊗ij}{\tilde{X}},\underset{⊗lj}{\tilde{X}}\right)+2\eta w_{1}+\xi_{1}=0\\ \frac{\partial L\left(w_{1},w_{2},\cdots ,w_{n},\eta ,\xi_{1},\xi_{2},\cdots ,\xi_{n}\right)}{\partial w_{2}}=\sum_{i=1}^{m}\sum_{l=1}^{m}d\left(\underset{⊗ij}{\tilde{X}},\underset{⊗lj}{\tilde{X}}\right)+2\eta w_{2}+\xi_{2}=0\\ \vdots \\ \frac{\partial L\left(w_{1},w_{2},\cdots ,w_{n},\eta ,\xi_{1},\xi_{2},\cdots ,\xi_{n}\right)}{\partial w_{p}}=\sum_{i=1}^{m}\sum_{l=1}^{m}d\left(\underset{⊗ij}{\tilde{X}},\underset{⊗lj}{\tilde{X}}\right)+2\eta w_{n}+\xi_{n}=0\\ \sum_{j=1}^{n}w_{j}^{2}-1=0,\xi_{1}w_{1}=0,\xi_{2}w_{2}=0,\cdots ,\xi_{n}w_{n}=0 \end{array}\right.$$

Solving these equations, we can get:

(40) $$w_{j}=\frac{\sum_{i=1}^{m}\sum_{l=1}^{m}d\left(\underset{⊗ij}{\tilde{X}},\underset{⊗lj}{\tilde{X}}\right)}{\sqrt{\sum_{j=1}^{n}{\left(\sum_{i=1}^{m}\sum_{l=1}^{m}d\left(\underset{⊗ij}{\tilde{X}},\underset{⊗lj}{\tilde{X}}\right)\right)}^{2}}}$$

And then, after normalization, we can get:

(41) $$w_{j}=\frac{w_{j}}{\sum_{j=1}^{n}w_{j}}=\frac{\frac{\sum_{i=1}^{m}\sum_{l=1}^{m}d\left(\underset{⊗ij}{\tilde{X}},\underset{⊗lj}{\tilde{X}}\right)}{\sqrt{\sum_{j=1}^{n}{\left(\sum_{i=1}^{m}\sum_{l=1}^{m}d\left(\underset{⊗ij}{\tilde{X}},\underset{⊗lj}{\tilde{X}}\right)\right)}^{2}}}}{\frac{\sum_{j=1}^{n}\sum_{i=1}^{m}\sum_{l=1}^{m}d\left(\underset{⊗ij}{\tilde{X}},\underset{⊗lj}{\tilde{X}}\right)}{\sqrt{\sum_{j=1}^{n}{\left(\sum_{i=1}^{m}\sum_{l=1}^{m}d\left(\underset{⊗ij}{\tilde{X}},\underset{⊗lj}{\tilde{X}}\right)\right)}^{2}}}}=\frac{\sum_{i=1}^{m}\sum_{l=1}^{m}d\left(\underset{⊗ij}{\tilde{X}},\underset{⊗lj}{\tilde{X}}\right)}{\sum_{j=1}^{n}\sum_{i=1}^{m}\sum_{l=1}^{m}d\left(\underset{⊗ij}{\tilde{X}},\underset{⊗lj}{\tilde{X}}\right)}$$

#### 4.2.3. Grey Relation Coefficient Matrix

(1) The positive/negative ideal solutions. Using Formulas (31) and (32), the group decision matrix $\underset{⊗}{\tilde{X}}={\left[\underset{⊗ij}{\tilde{X}}\right]}_{m\times n}$ was drawn. The representation of each element of the matrix is $\underset{⊗ij}{\tilde{X}}=\left(\left(s_{\alpha_{ij}^{X}},s_{\beta_{ij}^{X}},s_{\delta_{ij}^{X}},s_{\gamma_{ij}^{X}}\right),\left[g_{ij}^{L},g_{ij}^{U}\right]\right)$.

According to the group decision matrix, the positive ideal solution is ${\underset{⊗}{\tilde{V}}}^{+}=\left({\underset{⊗1}{\tilde{V}}}^{+},{\underset{⊗2}{\tilde{V}}}^{+},\cdot \cdot \cdot ,{\underset{⊗n}{\tilde{V}}}^{+}\right)$. The expression of every element of the positive ideal solution vector is IGTFLVs ${\underset{⊗j}{\tilde{V}}}^{+}=\left(\left(s_{\alpha_{j}^{V}}^{+},s_{\beta_{j}^{V}}^{+},s_{\delta_{j}^{V}}^{+},s_{\gamma_{j}^{V}}^{+}\right),\left[h_{j}^{+L},h_{j}^{+U}\right]\right)$, where

(42) $$\begin{array}{l} s_{\delta_{j}^{V}}^{+}=\underset{i}{\max}\left(s_{\delta_{ij}^{X}}\right),\;s_{\gamma_{j}^{V}}^{+}=\underset{i}{\max}\left(s_{\gamma_{ij}^{X}}\right),\\ h_{j}^{+L}=\underset{i}{\min}\left(g_{ij}^{L}\right),\;h_{j}^{+U}=\underset{i}{\min}\left(g_{ij}^{U}\right) \end{array}$$

Similarly, the negative ideal solution is ${\underset{⊗}{\tilde{V}}}^{-}=\left({\underset{⊗1}{\tilde{V}}}^{-},{\underset{⊗2}{\tilde{V}}}^{-},\cdot \cdot \cdot ,{\underset{⊗n}{\tilde{V}}}^{-}\right)$. The expression of every element of the negative ideal solution vector is IGTFLVs ${\underset{⊗j}{\tilde{V}}}^{-}=\left(\left(s_{\alpha_{j}^{V}}^{-},s_{\beta_{j}^{V}}^{-},s_{\delta_{j}^{V}}^{-},s_{\gamma_{j}^{V}}^{-}\right),\left[h_{j}^{-L},h_{j}^{-U}\right]\right)$, where

(43) $$s_{\alpha_{j}^{V}}^{-}=\underset{i}{\min}\left(s_{\alpha_{ij}^{X}}\right),\;s_{\beta_{j}^{V}}^{-}=\underset{i}{\min}\left(s_{\beta_{ij}^{X}}\right),\;s_{\delta_{j}^{V}}^{-}=\underset{i}{\min}\left(s_{\delta_{ij}^{X}}\right),\;s_{\gamma_{j}^{V}}^{-}=\underset{i}{\min}\left(s_{\gamma_{ij}^{X}}\right),\;h_{j}^{-L}=\underset{i}{\max}\left(g_{ij}^{L}\right),\;h_{j}^{-U}=\underset{i}{\max}\left(g_{ij}^{U}\right)$$

(2) Grey relation coefficient matrix based on the positive ideal solution

(44) $$\varepsilon_{ij}^{+}=\frac{s+\rho M}{\Delta_{ij}^{+}+\rho M}\;\rho \in \left(0,1\right)$$

where, $\Delta_{ij}^{+}=d\left({\underset{⊗ij}{\tilde{X}}}^{+},{\underset{⊗j}{\tilde{V}}}^{+}\right)$, $s=\underset{i}{\min}\;\underset{j}{\min}\;\Delta_{ij}^{+}$, $M=\underset{i}{\max}\;\underset{j}{\max}\;\Delta_{ij}^{+}$, ρ is the resolution factor, the general value of 0.5.

The positive grey relation coefficient matrix is:

(45) $$\varepsilon^{+}=\left[\begin{array}{cccc} \varepsilon_{11}^{+} & \varepsilon_{12}^{+} & \cdots & \varepsilon_{1n}^{+}\\ \varepsilon_{21}^{+} & \varepsilon_{22}^{+} & \cdots & \varepsilon_{2n}^{+}\\ \vdots & \vdots & \vdots & \vdots \\ \varepsilon_{m1}^{+} & \varepsilon_{m2}^{+} & \cdots & \varepsilon_{mn}^{+} \end{array}\right]$$

(3) Grey relation coefficient matrix based on the negative ideal solution

(46) $$\varepsilon_{ij}^{-}=\frac{s+\rho M}{\Delta_{ij}^{-}+\rho M}\;\rho \in \left(0,1\right)$$

where, $\Delta_{ij}^{-}=d\left({\underset{⊗ij}{\tilde{X}}}^{-},{\underset{⊗j}{\tilde{V}}}^{-}\right)$, $s=\underset{i}{\min}\;\underset{j}{\min}\;\Delta_{ij}^{-}$, $M=\underset{i}{\max}\;\underset{j}{\max}\;\Delta_{ij}^{-}$, ρ is the resolution factor, and the general value of 0.5.

The negative grey relation coefficient matrix is:

(47) $$\varepsilon^{-}=\left[\begin{array}{cccc} \varepsilon_{11}^{-} & \varepsilon_{12}^{-} & \cdots & \varepsilon_{1n}^{-}\\ \varepsilon_{21}^{-} & \varepsilon_{22}^{-} & \cdots & \varepsilon_{2n}^{-}\\ \vdots & \vdots & \vdots & \vdots \\ \varepsilon_{m1}^{-} & \varepsilon_{m2}^{-} & \cdots & \varepsilon_{mn}^{-} \end{array}\right]$$

#### 4.2.4. Sort the Alternatives

The positive grey relational grade is: $R_{i}^{+}=\sum_{j}^{n}w_{j}\varepsilon_{ij}^{+},\left(i=1,2,\cdots ,m\right)$. The negative grey relational grade is: $R_{i}^{-}=\sum_{j}^{n}w_{j}\varepsilon_{ij}^{-},\left(i=1,2,\cdots ,m\right)$. The relative closeness of the grey relation of each scheme is

(48) $$C_{i}=\frac{R_{i}^{+}}{R_{i}^{+}+R_{i}^{-}},\left(i=1,2,\cdots ,m\right)$$

From the grey relation axiom it can be seen that any two sequences of behavior have a certain relevance in the system, that is $0<R_{i}^{+}\leq 1$, $0<R_{i}^{-}\leq 1$, so the grey relation relative degree is $0<C_{i}<1$. If $\max\left(C_{1},C_{2},\cdots ,C_{m}\right)=C_{i}$, the *i*th alternative is best.

## 5. Examples

Problem Description: Assume that the three experts $\left\{e_{1},e_{2},e_{3}\right\}$ assess the sewage-treatment capacity of the four companies $\left\{A_{1},A_{2},A_{3},A_{4}\right\}$. Because this paper presents a simulation case, the sewage-treatment capacity is assessed from only four aspects (what we call attributes). Namely: sewage-treatment equipment $\left(C_{1}\right)$, financial strength $\left(C_{2}\right)$, sewage-management capacity $\left(C_{3}\right)$, sewage-treatment procedures $\left(C_{4}\right)$. In this paper, the weights of experts and attributes are unknown and need to be estimated using the proposed method. Of course, it can be subjectively given as in some other papers. Each expert uses the IGTFLVs to evaluate the attribute value, as shown in Table 1, Table 2 and Table 3. Let $S=\left(s_{0},s_{1},s_{2},s_{3},s_{4},s_{5},s_{6},s_{7},s_{8}\right)$ be the linguistic label. The expert weights are first calculated and the evaluation information in Table 1, Table 2 and Table 3 is integrated into the group evaluation information. Then, we calculate the attribute weight. Finally, we use the improved grey relation analysis method to obtain the representative value of each company. In this way, we can draw the size of the wastewater treatment capacity of the four companies and select the ones with the strongest treatment capacity. The sewage treatment capacity of four enterprises is evaluated, and the specific evaluation process is as follows:

### 5.1. Decision-Making Steps

(1) According to the above conditions, the expert weights $\lambda =\left(\lambda_{1},\lambda_{2},\cdots ,\lambda_{p}\right)$ and group decision matrix $\underset{⊗}{\tilde{X}}$ are derived based on Formulas (31)–(41). The reason for using formula (34)–(36) is to find the right expert weight so that the evaluation matrix and the group evaluation matrix of each expert are the closest. So we can draw:

λ=(0.30802,0.30721,0.38477). Through the results we can see that the evaluation of $e_{1}$ and $e_{2}$ are closer, and the evaluation of $e_{3}$ deviates from the high. So the result of the group decision matrix is as follows:

(49) $$\begin{array}{c} \underset{⊗}{\tilde{X}}=[\begin{array}{ll} \left(\left(s_{1.92},s_{2.92},s_{4.54},s_{5.54}\right),\left[0.42,0.71\right]\right) & \left(\left(s_{1.00},s_{2.00},s_{3.69},s_{4.69}\right),\left[0.66,0.71\right]\right)\\ \left(\left(s_{2.31},s_{3.3},s_{5.31},s_{6.31}\right),\left[0.75,0.79\right]\right) & \left(\left(s_{1.62},s_{2.62},s_{4.23},s_{5.62}\right),\left[0.71,0.82\right]\right)\\ \left(\left(s_{1.31},s_{2.31},s_{3.92},s_{5.31}\right),\left[0.49,0.71\right]\right) & \left(\left(s_{1.62},s_{2.62},s_{4.62},s_{6.00}\right),\left[0.55,0.71\right]\right)\\ \left(\left(s_{2.54},s_{3.54},s_{5.54},s_{6.54}\right),\left[0.72,0.83\right]\right) & \left(\left(s_{1.31},s_{2.31},s_{4.00},s_{5.00}\right),\left[0.57,0.72\right]\right) \end{array}\to \\ \to \begin{array}{ll} \left(\left(s_{2.00},s_{3.00},s_{5.00},s_{6.00}\right),\left[0.76,0.80\right]\right) & \left(\left(s_{2.31},s_{3.31},s_{5.31},s_{6.31}\right),\left[0.62,0.79\right]\right)\\ \left(\left(s_{1.31},s_{2.31},s_{3.92},s_{4.92}\right),\left[0.35,0.62\right]\right) & \left(\left(s_{1.31},s_{2.31},s_{4.31},s_{5.31}\right),\left[0.64,0.72\right]\right)\\ \left(\left(s_{0.92},s_{1.92},s_{3.23},s_{4.23}\right),\left[0.62,0.66\right]\right) & \left(\left(s_{2.00},s_{3.00},s_{4.62},s_{5.62}\right),\left[0.55,0.66\right]\right)\\ \left(\left(s_{1.00},s_{2.00},s_{2.92},s_{3.92}\right),\left[0.61,0.78\right]\right) & \left(\left(s_{1.31},s_{2.69},s_{4.31},s_{5.31}\right),\left[0.66,0.82\right]\right) \end{array}] \end{array}$$

The same can be obtained. The attribute weights are derived based on Formulas (37)–(41).

(50) w=(0.25551,0.20986,0.2972,0.23743)

(2) The ideal solution is calculated by Formulas (42) and (43).

(51) $$\begin{array}{l} {\underset{⊗}{\tilde{V}}}^{+}=(\begin{array}{cc} \left(\left(s_{2.54},s_{3.54},s_{5.54},s_{6.54}\right),\left[0.42,0.71\right]\right) & \left(\left(s_{1.62},s_{2.62},s_{4.62},s_{6.00}\right),\left[0.55,0.71\right]\right) \end{array}\\ \begin{array}{cc} \left(\left(s_{2.00},s_{3.00},s_{5.00},s_{6.00}\right),\left[0.35,0.62\right]\right) & \left(\left(s_{2.31},s_{3.31},s_{5.31},s_{6.31}\right),\left[0.55,0.66\right]\right) \end{array})\\ {\underset{⊗}{\tilde{V}}}^{-}=(\begin{array}{cc} \left(\left(s_{1.31},s_{2.31},s_{3.92},s_{5.31}\right),\left[0.75,0.83\right]\right) & \left(\left(s_{1.00},s_{2.00},s_{3.69},s_{4.69}\right),\left[0.71,0.82\right]\right) \end{array}\\ \begin{array}{cc} \left(\left(s_{0.92},s_{1.92},s_{2.92},s_{3.92}\right),\left[0.76,0.80\right]\right) & \left(\left(s_{1.31},s_{2.31},s_{4.31},s_{5.31}\right),\left[0.66,0.82\right]\right) \end{array}) \end{array}$$

(3) According to Formulas (45) and (46), we can obtain $\varepsilon^{+}$ and $\varepsilon^{-}$.

(52) $$\varepsilon^{+}=\left[\begin{array}{cccc} 0.6521 & 0.5780 & 0.3573 & 0.6082\\ 0.4020 & 0.5481 & 0.5902 & 0.5053\\ 0.4918 & 1.0000 & 0.3652 & 0.7698\\ 0.4074 & 0.7212 & 0.3334 & 0.4438 \end{array}\right]\;\varepsilon^{-}=\left[\begin{array}{cccc} 0.3782 & 0.7402 & 0.6355 & 0.5836\\ 0.6550 & 0.8063 & 0.3487 & 0.7569\\ 0.4857 & 0.4533 & 0.6080 & 0.4721\\ 0.6389 & 0.5703 & 0.7468 & 1.0000 \end{array}\right]$$

### 5.2. Rank the Alternatives

So the result of the positive/negative grey relational grade as follows:

(53) $$R^{+}=\left(0.5385,0.5131,0.6268,0.4599\right)\;R^{-}=\left(0.5794,0.6199,0.5120,0.7423\right)$$

Therefore, the relative closeness of the grey relation of each scheme is:

(54) C=(0.4817,0.4529,0.5504,0.3825)

So the four companies $\left\{A_{1},A_{2},A_{3},A_{4}\right\}$ of the sewage-treatment capacity assessment results are as follows: $A_{3}≻A_{1}≻A_{2}≻A_{4}$.

### 5.3. Compared with Other Method

IGTFLV can be arbitrarily converted into interval grey linguistic variables, interval grey uncertainty language variables, trapezoidal fuzzy linguistic variables, uncertain fuzzy linguistic variables, and so on. Therefore, IGTFLV can be regarded as an extension of the previous model, which is more inclusive.

(55) $$\begin{array}{l} \mathrm{If}\;s_{\alpha }=s_{\beta }=s_{\delta }=s_{\gamma },\;\mathrm{then}\;\left(\left[s_{\alpha },s_{\beta },s_{\delta },s_{\gamma }\right],\left[g^{L},g^{U}\right]\right)\Leftrightarrow \left(s_{\alpha },\left[g^{L},g^{U}\right]\right)\\ \mathrm{If}\;s_{\alpha }=s_{\beta }<s_{\delta }=s_{\gamma },\;then\;\left(\left[s_{\alpha },s_{\beta },s_{\delta },s_{\gamma }\right],\left[g^{L},g^{U}\right]\right)\Leftrightarrow \left(\left[s_{\alpha },s_{\delta }\right],\left[g^{L},g^{U}\right]\right)\\ \mathrm{If}\;g^{L}=g^{U}=0,\;\mathrm{then}\;\left(\left[s_{\alpha },s_{\beta },s_{\delta },s_{\gamma }\right],\left[g^{L},g^{U}\right]\right)\Leftrightarrow \left[s_{\alpha },s_{\beta },s_{\delta },s_{\gamma }\right]\\ \mathrm{If}\;s_{\alpha }=s_{\beta }=s_{\delta }=s_{\gamma }\;\mathrm{and}\;g^{L}=g^{U}=0,\;\mathrm{then}\;\left(\left[s_{\alpha },s_{\beta },s_{\delta },s_{\gamma }\right],\left[g^{L},g^{U}\right]\right)\Leftrightarrow s_{\alpha }\\ \mathrm{If}\;s_{\alpha }=s_{\beta }<s_{\delta }=s_{\gamma }\;\mathrm{and}\;g^{L}=g^{U}=0,\;\mathrm{then}\;\left(\left[s_{\alpha },s_{\beta },s_{\delta },s_{\gamma }\right],\left[g^{L},g^{U}\right]\right)\Leftrightarrow \left[s_{\alpha },s_{\delta }\right] \end{array}$$

(1) Using the above method to calculate the case of the paper [39].

The expert weight used in the paper [39] is (0.4,0.32,0.28), the attribute weight is (0.34,0.22,0.23,0.21), and the result is $A_{3}≻A_{1}≻A_{2}≻A_{4}$.

This paper uses the weight of the paper [39], and the result is $A_{3}≻A_{1}≻A_{2}≻A_{4}$. The results show that, if we continue to use the weight of the paper [39], then the results obtained using this method will be consistent with the original.

If the weights are unknown, the expert weight obtained in this paper is (0.332,0.333,0.335), and the attribute weight obtained in this paper is (0.348,0.224,0.235,0.194). The result of using the improved grey relational analysis method is $A_{3}≻A_{1}≻A_{2}≻A_{4}$.

(2) Using the above method to calculate the case of the paper [40].

The case used in the paper of [40] is derived from the paper [41]. If the paper [40] uses the attribute weights (0.3,0.2,0.1,0.4) in the paper [41], the result is $A_{4}≻A_{2}≻A_{3}≻A_{1}$. If the paper [40] uses its own calculated attribute weight (0.253,0.302,0.157,0.288), the result is $A_{4}≻A_{2}≻A_{1}≻A_{3}$.

If this paper uses the attribute weights in the paper [41], the result is $A_{4}≻A_{2}≻A_{3}≻A_{1}$. If this paper uses the attribute weights in the paper [40], the result is $A_{4}≻A_{2}≻A_{1}≻A_{3}$.

The attribute weight obtained in this paper is (0.256,0.319,0.188,0.238), and the result is $A_{4}≻A_{2}≻A_{1}≻A_{3}$. Obviously, the results obtained in this paper are consistent with the paper [40,41]. Although the weight obtained in this paper is inconsistent with the original, this is because the two methods have different principles.

(3) Using the above method to calculate the case of the paper [42].

The expert weight used in the paper [42] is (0.4,0.32,0.28), the attribute weight is (0.3,0.2,0.2,0.3).

The paper [42] used different w and λ, and the result is only two cases $A_{3}≻A_{1}≻A_{2}≻A_{4}$ and $A_{3}≻A_{2}≻A_{1}≻A_{4}$.

This paper uses the weight of the paper [42], and the result is $A_{3}≻A_{1}≻A_{2}≻A_{4}$.

If the weights are unknown, the expert weight obtained in this paper is (0.322,0.334,0.344), and the attribute weight obtained in this paper is (0.346,0.249,0.211,0.194). The result of using the improved grey relational analysis method is $A_{3}≻A_{1}≻A_{2}≻A_{4}$.

## 6. Conclusions

Multi-attribute group decision making (MAGDM) usually contains the effects of subjective and objective factors. Therefore, this kind of problem should include two parts: grey and fuzzy. Previously, the expressions of fuzzy information have included linguistic variables, interval numbers, uncertain linguistic variables, triangular fuzzy numbers and trapezoidal fuzzy numbers. The actual situation chooses a more suitable fuzzy expression. For example, when we obtain more information, we choose a linguistic variable or interval number to express fuzzy information. When we obtain less information, we choose an uncertain linguistic variable or trapezoidal fuzzy number to express fuzzy information. In this paper, we extend the grey fuzzy number model by putting forward interval grey trapezoid linguistic variables. IGTFLVs are more inclusive and flexible. The IGTFLV can not only contain more fuzziness information, but also simplify the model at any time according to the given situation. In this paper, the attribute weights are also determined by the maximum deviation method. The method of determining the experts weight also comes from the maximum deviation method. However, the difference is that this paper uses the maximum similarity method. The deviation minimization of group evaluation and individual evaluation is equivalent to similarity maximization. Therefore, this paper uses the projection value between IGTFLV vectors to express the similarity between group evaluation and individual evaluation. This paper also maximizes total similarity in order to obtain expert weights. Finally, the grey relational decision-making method was used to evaluate the wastewater-treatment capacity of four enterprises. The results show that IGTFLVs are highly inclusive and flexible, and the improved grey relational analysis method is more effective and accurate. In practice, more real data units must be obtained and verified one by one. In future, we will study how to obtain data and apply the methods of this article to more fields.

## Figures and Tables

**Table 1:** The attribute values of each attribute with respect to four enterprises given by expert $e_{1}$.

Enterprises	Attribute ($C_{1}$)	Attribute ($C_{2}$)	Attribute ($C_{3}$)	Attribute ($C_{4}$)
$A_{1}$	$\left(\left[s_{3},s_{4},s_{6},s_{7}\right],\left[0.2,0.3\right]\right)$	$\left(\left[s_{1},s_{2},s_{3},s_{4}\right],\left[0.4,0.4\right]\right)$	$\left(\left[s_{3},s_{4},s_{6},s_{7}\right],\left[0.5,0.5\right]\right)$	$\left(\left[s_{1},s_{2},s_{4},s_{5}\right],\left[0.2,0.4\right]\right)$
$A_{2}$	$\left(\left[s_{2},s_{3},s_{5},s_{6}\right],\left[0.4,0.4\right]\right)$	$\left(\left[s_{3},s_{4},s_{6},s_{7}\right],\left[0.4,0.5\right]\right)$	$\left(\left[s_{1},s_{2},s_{4},s_{5}\right],\left[0.1,0.2\right]\right)$	$\left(\left[s_{2},s_{3},s_{5},s_{6}\right],\left[0.5,0.5\right]\right)$
$A_{3}$	$\left(\left[s_{1},s_{2},s_{4},s_{5}\right],\left[0.2,0.3\right]\right)$	$\left(\left[s_{2},s_{3},s_{5},s_{6}\right],\left[0.2,0.3\right]\right)$	$\left(\left[s_{2},s_{3},s_{5},s_{6}\right],\left[0.3,0.3\right]\right)$	$\left(\left[s_{3},s_{4},s_{6},s_{7}\right],\left[0.2,0.3\right]\right)$
$A_{4}$	$\left(\left[s_{4},s_{5},s_{7},s_{8}\right],\left[0.5,0.6\right]\right)$	$\left(\left[s_{1},s_{2},s_{3},s_{4}\right],\left[0.2,0.2\right]\right)$	$\left(\left[s_{1},s_{2},s_{4},s_{5}\right],\left[0.2,0.4\right]\right)$	$\left(\left[s_{1},s_{2},s_{4},s_{5}\right],\left[0.3,0.4\right]\right)$

**Table 2:** The attribute values of each attribute with respect to four enterprises given by expert $e_{2}$.

Enterprises	Attribute ($C_{1}$)	Attribute ($C_{2}$)	Attribute ($C_{3}$)	Attribute ($C_{4}$)
$A_{1}$	$\left(\left[s_{2},s_{3},s_{5},s_{6}\right],\left[0.1,0.3\right]\right)$	$\left(\left[s_{1},s_{2},s_{4},s_{5}\right],\left[0.2,0.3\right]\right)$	$\left(\left[s_{1},s_{2},s_{4},s_{5}\right],\left[0.2,0.2\right]\right)$	$\left(\left[s_{4},s_{5},s_{7},s_{8}\right],\left[0.4,0.5\right]\right)$
$A_{2}$	$\left(\left[s_{3},s_{4},s_{6},s_{7}\right],\left[0.4,0.5\right]\right)$	$\left(\left[s_{1},s_{2},s_{4},s_{5}\right],\left[0.3,0.4\right]\right)$	$\left(\left[s_{2},s_{3},s_{5},s_{6}\right],\left[0.2,0.4\right]\right)$	$\left(\left[s_{1},s_{2},s_{4},s_{5}\right],\left[0.2,0.3\right]\right)$
$A_{3}$	$\left(\left[s_{2},s_{3},s_{5},s_{6}\right],\left[0.2,0.4\right]\right)$	$\left(\left[s_{2},s_{3},s_{5},s_{6}\right],\left[0.2,0.3\right]\right)$	$\left(\left[s_{1},s_{2},s_{3},s_{4}\right],\left[0.4,0.4\right]\right)$	$\left(\left[s_{1},s_{2},s_{4},s_{5}\right],\left[0.3,0.3\right]\right)$
$A_{4}$	$\left(\left[s_{3},s_{4},s_{6},s_{7}\right],\left[0.3,0.4\right]\right)$	$\left(\left[s_{2},s_{3},s_{5},s_{6}\right],\left[0.4,0.5\right]\right)$	$\left(\left[s_{1},s_{2},s_{3},s_{4}\right],\left[0.3,0.4\right]\right)$	$\left(\left[s_{2},s_{3},s_{5},s_{6}\right],\left[0.2,0.4\right]\right)$

**Table 3:** The attribute values of each attribute with respect to four enterprises given by expert $e_{3}$.

Enterprises	Attribute ($C_{1}$)	Attribute ($C_{2}$)	Attribute ($C_{3}$)	Attribute ($C_{4}$)
$A_{1}$	$\left(\left[s_{1},s_{2},s_{3},s_{4}\right],\left[0.2,0.4\right]\right)$	$\left(\left[s_{1},s_{2},s_{4},s_{5}\right],\left[0.3,0.3\right]\right)$	$\left(\left[s_{2},s_{3},s_{5},s_{6}\right],\left[0.4,0.5\right]\right)$	$\left(\left[s_{2},s_{3},s_{5},s_{6}\right],\left[0.2,0.3\right]\right)$
$A_{2}$	$\left(\left[s_{2},s_{3},s_{5},s_{6}\right],\left[0.3,0.3\right]\right)$	$\left(\left[s_{1},s_{2},s_{3},s_{5}\right],\left[0.3,0.4\right]\right)$	$\left(\left[s_{1},s_{2},s_{3},s_{4}\right],\left[0.1,0.2\right]\right)$	$\left(\left[s_{1},s_{2},s_{4},s_{5}\right],\left[0.1,0.2\right]\right)$
$A_{3}$	$\left(\left[s_{1},s_{2},s_{3},s_{5}\right],\left[0.2,0.3\right]\right)$	$\left(\left[s_{1},s_{2},s_{4},s_{6}\right],\left[0.3,0.4\right]\right)$	$\left(\left[s_{0},s_{1},s_{2},s_{3}\right],\left[0.1,0.2\right]\right)$	$\left(\left[s_{2},s_{3},s_{4},s_{5}\right],\left[0.2,0.3\right]\right)$
$A_{4}$	$\left(\left[s_{1},s_{2},s_{4},s_{5}\right],\left[0.2,0.3\right]\right)$	$\left(\left[s_{1},s_{2},s_{4},s_{5}\right],\left[0.1,0.3\right]\right)$	$\left(\left[s_{1},s_{2},s_{2},s_{3}\right],\left[0.3,0.4\right]\right)$	$\left(\left[s_{1},s_{3},s_{4},s_{5}\right],\left[0.4,0.5\right]\right)$

## References

[1] Chen W.K., Lin H.L. (2016). Research on decision-making of post-disaster reconstruction scheme for subway construction based on prospect theory. J. Saf. Sci. Technol..

[2] Sun P.A., Tan Q.Y. (2016). On text information mining technology based on multi-attribute decision theory. J. Southwest China Norm. Univ. (Nat. Sci. Ed.).

[3] Zhang H.Y., Wen F.S., Zhang C., Tian C.Z. (2016). Prospect theory based multiple-attribute decision-making method for determining portfolio of construction projects in power systems. Autom. Electr. Power Syst..

[4] Zhang L.P., Yu Z.J., Li W.C. (2016). Application of multiple attribute decision making in quality assessment on maternal and children health care. Chin. J. Health Educ..

[5] Li C.X., Zhou Y., Lin H. (2016). Model of temporal spatial information fusion for BM. Mod. Def. Technol..

[6] Bu G.Z., Zhang Y.W. (2002). Grey fuzzy comprehensive evaluation based on the theory of grey fuzzy relation. Syst. Eng. Theory Pract..

[7] Zhu S.Q., Meng K., Zhang H.X. (2006). Interval numbers grey fuzzy comprehensive evaluation and its application. Electron. Opt. Control.

[8] Wang J.Q., Wang J. (2008). Interval grey fuzzy multi-criteria decision making approach. Syst. Eng. Electron..

[9] Hu L.F., Guan X., He Y. (2012). A new approach for grey multi-attribute decision making. Control Decis..

[10] Cao G., Shen L.X. (2014). A multiple attribute group decision making model based on three-parameter interval grey linguistic variable. Oper. Res. Manag. Sci..

[11] Wang H.D., Liu P.D., Li C.D., Zhang X., Liu W.L. (2014). A multi-attribute group decision making method based on interval grey trapezoidal fuzzy numbers. J. Inner Mongolia Univ. Nat. Sci. Ed..

[12] Bordogna G., Fedrizzi M., Pasi G. (1997). A linguistic modeling of consensus in group decision making based on OWA operators. IEEE Trans. Syst. Man Cybern. Part A Syst. Hum..

[13] Liu P.D., Zhang X. (2011). Multi-attribute group decision making method based on interval grey linguistic variables weighted geometric aggregation operator. Control Decis..

[14] Liu P. (2013). The multi-attribute group decision making method based on the interval grey linguistic variables weighted aggregation operator. J. Intell. Fuzzy Syst..

[15] Jin F., Liu P., Zhang X. (2013). The multi-attribute group decision making method based on the interval grey linguistic variables weighted harmonic aggregation operators. Technol. Econ. Dev. Econ..

[16] Wei G.W. (2011). Grey relational analysis method for 2-tuple linguistic multiple attribute group decision making with incomplete weight information. Expert Syst. Appl..

[17] Ma Z.J., Zhang N., Dai Y. (2013). Some induced correlated aggregating operators with interval grey uncertain linguistic information and their application to multiple attribute group decision making. Math. Probl. Eng..

[18] Han E.D., Guo P., Zhao J. (2015). Method for multi-attribute group decision making based on interval grey uncertain linguistic information. Comput. Eng. Appl..

[19] Jin F., Liu P. (2012). A multi-attribute group decision-making method based on weighted geometric aggregation operators of interval-valued trapezoidal fuzzy numbers. Appl. Math. Model..

[20] Liu S.F., Dang Y.G., Fan Z.G. (2010). Grey System Theory and Its Application.

[21] Luo D., Liu S.F. (2005). Study on the method for grey incidence decision-making. Chin. J. Manag. Sci..

[22] Luo D., Liu S.F. (2005). Grey incidence decision-making with incomplete information. J. Appl. Sci..

[23] Sun X.D., Jiao Y., Hu J.S. (2005). Research on decision-making method based on grey relation degree and TOPSIS. Chin. J. Manag. Sci..

[24] Luo D. (2009). Decision-making methods with three-parameter interval grey number. Syst. Eng. Theory Pract..

[25] Wang X.J., Zhang Y. (2011). Non-uniform grey relational method based on AHP and DEA. Syst. Eng. Theory Pract..

[26] Jiang S.Q., Liu S.F., Liu Z.X., Fang Z.G. (2015). Grey incidence decision making model based on area. Control Decis..

[27] Yin K., Zhang Y., Li X. (2017). Research on storm-tide disaster losses in China using a new grey relational analysis model with the dispersion of panel data. Int. J. Environ. Res. Public Health.

[28] Wang Y.M. (1998). Using the method of maximizing deviations to make decision for multi-indices. Syst. Eng. Electron..

[29] Wu Z.B., Chen Y.H. (2007). The maximizing deviation method for group multiple attribute decision making under linguistic environment. Fuzzy Sets Syst..

[30] Bapi D., Guha D. (2015). Partitioned Bonferroni mean based on linguistic 2-tuple for dealing with multi-attribute group decision making. Appl. Soft Comput..

[31] Wang C.H., Song L.T. (1988). Fuzzy Theory and Methodology.

[32] Chen D.W. (1994). Grey Fuzzy Set Introduction.

[33] Li H.X., Wang P.Z. (1994). Fuzzy Mathematics.

[34] Wang Q.Y. (1996). Grey Fuzzy Mathematical Foundation.

[35] Herrera F., Herrera-Viedma E. (2000). Linguistic decision analysis: Steps for solving decision problems under linguistic information. Fuzzy Sets Syst..

[36] Herrera F., Viedma E.H., Verdegay J.L. (1996). A model of consensus in group decision making under linguistic assessments. Fuzzy Sets Syst..

[37] Xu Z.S. (2006). A note on linguistic hybrid arithmetic averaging operator in multiple attribute group decision making with linguistic information. Group Decis. Negot..

[38] Xu Z.S. (2006). Goal programming models for multiple attribute decision making under linguistic setting. J. Manag. Sci. China.

[39] Jin F., Liu P.D. (2010). The multi-attribute group decision making method based on the interval grey linguistic variables. Afr. J. Bus. Manag..

[40] Han Z.S., Liu P.D. (2011). Multiple attribute decision making method based on trapezoid fuzzy linguistic variables. Fuzzy Syst. Math..

[41] Liang X.C., Chen S.F. (2008). Multiple attribute decision making method based on trapezoid fuzzy linguistic variables. J. Southeast Univ. (Engl. Ed.).

[42] Liu P., Chu Y., Li Y. (2015). The multi-attribute group decision-making method based on the interval grey uncertain linguistic generalized hybrid averaging operator. Neural Comput. Appl..

